# Chemopreventive Effects of Strawberry and Black Raspberry on Colorectal Cancer in Inflammatory Bowel Disease

**DOI:** 10.3390/nu11061261

**Published:** 2019-06-03

**Authors:** Tong Chen, Ni Shi, Anita Afzali

**Affiliations:** 1 Division of Medical Oncology, Department of Internal Medicine, The Ohio State University, Columbus, OH 43210, USA; ni.shi@osumc.edu; 2 Comprehensive Cancer Center, The Ohio State University, Columbus, OH 43210, USA; 3 Division of Gastroenterology, Hepatology and Nutrition, The Ohio State University, Columbus, OH 43210, USA; Anita.Afzali@osumc.edu; 4 Inflammatory Bowel Disease Center, Wexner Medical Center, The Ohio State University, Columbus, OH 43210, USA

**Keywords:** colorectal cancer, inflammatory bowel disease, colorectal cancer, dysplasia, berries, chemoprevention

## Abstract

Colorectal cancer (CRC) remains the third most common cause of cancer-related death in the United States and the fourth globally with a rising incidence. Inflammatory bowel disease (IBD) is a chronic immunologically mediated disease that imposes a significant associated health burden, including the increased risk for colonic dysplasia and CRC. Carcinogenesis has been attributed to chronic inflammation and associated with oxidative stress, genomic instability, and immune effectors as well as the cytokine dysregulation and activation of the nuclear factor kappa B (NFκB) signaling pathway. Current anti-inflammation therapies used for IBD treatment have shown limited effects on CRC chemoprevention, and their long-term toxicity has limited their clinical application. However, natural food-based prevention approaches may offer significant cancer prevention effects with very low toxicity profiles. In particular, in preclinical and clinical pilot studies, strawberry and black raspberry have been widely selected as food-based interventions because of their potent preventive activities. In this review, we summarize the roles of strawberry, black raspberry, and their polyphenol components on CRC chemoprevention in IBD.

## 1. Introduction

Inflammatory bowel disease (IBD) (e.g., Crohn’s disease and ulcerative colitis) is a chronic immunologically mediated disease that develops in interactions between genetics, immunology, the environment, and the microbiome [1,2]. IBD is highly prevalent in North America and Europe, affecting about 1.3 million people in the United States and 2.2 million people in Europe [1]. Moreover, the rising prevalence of IBD has been reported across other countries worldwide. Notably, patients with IBD are at the increased risk of developing colorectal cancer (CRC), which further increases the urgency of understanding and treating this disease [3]. 

In IBD, chronic inflammation induces oncogenic mutations, genomic instability, immune microenvironment changes, early tumor promotion, and angiogenesis. These factors are likely related to the increased risk of CRC in IBD [4]. Importantly, supporting the association between chronic inflammation in IBD and CRC risk, the results of studies employing 5-aminosalicylate (5-ASAs) to target and reduce inflammation identified some chemopreventive effects in IBD [5]. In population-based cohorts, the incidence of CRC in patients with ulcerative colitis was 2.4-fold higher than in the controls [6]. Furthermore, some results suggested that at 10 years after the initial onset of ulcerative colitis around 2% of patients with ulcerative colitis developed CRC: 8% after 20 years and 18% after 30 years of disease duration [7]. Previous data also showed that 2.9% of Crohn’s disease patients developed CRC within 10 years after the initial onset of the disease, and that percentage increased to 5.6% after 20 years and 8.3% after 30 years. Due to multifocal tumors and cell histopathology, CRC that develops in IBD patients tends to have a worse prognosis and survival rate compared to sporadic CRC in the advanced stage [8]. Additionally, the mean age for developing CRC in IBD patients (40–50 years) tends to be lower than the mean age for sporadic CRC (60 years) [4].

Due to the increased risk and poorer prognosis associated with CRC in individuals with IBD, its early detection and prevention are essential. In adults aged 50–75 years with an average risk of CRC, the US Preventive Services Task Force recommends yearly CRC screening by a fecal occult blood test (FOBT), sigmoidoscopy every five years, and colonoscopy every 10 years. In patients with IBD, a colonoscopy is recommended every one to two years beginning at 8–10 years from the diagnosis of the disease as well as multiple colonic biopsy specimens for dysplasia detection to screen for dysplasia or CRC [9]. Although chemoprevention is another important goal for IBD-related CRC management, the current therapies used in IBD have shown little to no effect, and the long-term toxicity associated with these treatments limits their clinical application for this purpose.

Diets that are high in animal fat and low in fruit and vegetables can be important triggers of both IBD and cancers [10,11]. Based on the associations between diet and cancers, natural food-based prevention approaches may represent a promising strategy. Notably, food-based approaches involving components of fruits and vegetables, vitamins, minerals, probiotics, and herbal medicines have shown potential cancer prevention effects with low toxicity profiles. Flavonoids, which are a major class of polyphenols that occur naturally in fruit and vegetables, have shown promising in vitro anti-colon cancer effects. Additionally, several case-control and cohort studies have verified the inverse association between flavonoid intake and CRC [12,13,14]. In particular, berries are a good source of polyphenols, especially anthocyanins, micronutrients, and fiber, which suggests that these foods may be associated with benefits for cardiovascular and immune health as well as chemoprevention [15]. Moreover, strawberry and black raspberry have been widely selected for food-based interventions in preclinical and pilot clinical studies because of their potent activity in the prevention of oral, esophageal, colon, skin, and prostate cancer [16,17,18,19,20]. Strawberry is the most commonly consumed berry type on the current market. Our previous study showed the significant chemoprevention effects of strawberry in CRC mouse model and its role in inflammation inhibition [18]. We also demonstrate that black raspberry inhibits oxidative stress, the inflammation mediators, cyclooxygenase-2 (COX-2) and inducible nitric oxide synthase (iNOS) and exhibits superior anti-cancer effects than inhibitors of COX-2 and iNOS in an animal model [21,22]. In this review, we summarize the roles of strawberry, black raspberry, and their polyphenol components on CRC chemoprevention in IBD.

## 2. Animal Models of Inflammatory Bowel Disease and IBD-Related Colorectal Cancer

Genetic engineering models can produce IBD in animals, such as muc-2 deficiency, muc-2/C3GnT dual deletion, muc-2/Core 1-derived O-glycans deficiency, P-gp deficiency, T-bet knockout, IKK-γ or IKK-β deletion, STAT3 deficiency, STAT3/IL-22 dual deficiency, XBP1 deletion, IL-10 deficiency, and IL-7 overexpression [2,11]. These targeted genes are responsible for epithelial barrier function, intestinal permeability, oxidative stress, cytokine, inflammation and immune effector regulation signaling pathway, and gut microflora etc. Additionally, colonic epithelium injures, ectogenic immunogenic response, adoptive transfer of naïve CD4^+^ T cells to immune deficiency animals are strategies to induce IBD in animals. In Dextran sulfate sodium (DSS) model, DSS in drinking water is toxic in the epithelial lining of the colon and produce severe colitis. DSS treatment combined with gene deficiency in immune effectors regulation, cytokine response and immune response signaling pathways can enhance the inflammation in animals. Intrarectal injection of the haptenating agent 2,4,6-trinitrobenzene sulfonic acid (TNBS) or 4-ethoxymethylene-2-phenyl-2-oxazolin-5-one (oxazolone) can cause ulcerative colitis with different features. The inflammation in the TNBS model is regulated by TH1-mediated immune response with CD4^+^ T cells, neutrophils and macrophages infiltration showing symptoms including severe diarrhea, weight loss, and rectal prolapse similar to Crohn’s disease. TNBS model is used for the immune-related studies for Crohn’s disease. The oxazolone model driven by NKT cells and IL-13 is considered as a valuable model for ulcerative colitis. The regulatory T cell deficiency in naïve CD4^+^ T cells is a critical factor for inflammation in the transfer model. This model is ideal for immunoregulation and regulatory T cell research.

**Figure 1:**
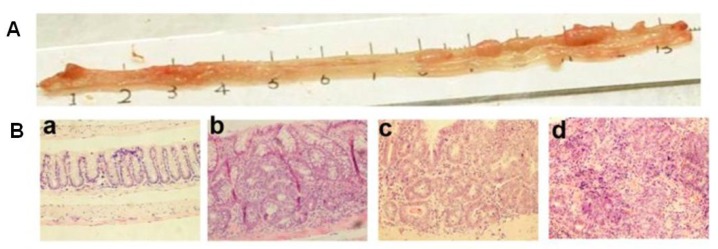
Histopathology of azoxymethane (AOM)/dextran sodium sulfate (DSS)-induced colorectal cancer (CRC) in mice. (**A**) The representative macroscopic appearance of a mouse colon treated with AOM/DSS; (**B**) The representative hematoxylin and eosin stained sections showing different histopathology of mouse colon, including normal (**a**), dysplasia (**b**), adenoma (**c**), and adenocarcinoma (**d**).

In studies on colon carcinogenesis and chemopreventive screening interventions for CRC in IBD, gene engineering mouse model such as apc mutation, apc deletion, smad mutation, smad 3 knockout, rag2-deficiency, k-ras mutation have been used [23]. In chemically induced preclinical models, murine treated with azoxymethane (AOM)/DSS has been a commonly used model for IBD-related CRC research for over a decade. AOM is a metabolite of 1,2-dimethylhydrazine (DMH) and the carcinogen to induce CRC in murine. It is important to note that AOM-induced tumors showed histopathological characteristics similar to human CRC, such as frequent K-Ras, β-catenin mutation, and microsatellite instability [24]. When combined with AOM, DSS works as a promoter of colorectal carcinogenesis through an initial acute inflammation phase. To induce colon carcinogenesis in murine, a single intraperitoneal injection of AOM at 10 mg/kg body weight or less can be given, followed by one to three cycles of 2% or 3% DSS in drinking water. In this method, tumors typically form 14 weeks after the AOM injection. Multistep tumor histogenesis can be efficiently reproduced in the AOM/DSS murine model. As found in our former studies, intraepithelial neoplasia, including changes ranging from dysplasia to adenoma and adenocarcinoma, were observed (Figure 1) [18]. T lymphocytes and other immune cells typically found in CRC patients are also frequently observed in tumors developed in AOM/DSS murine models. Therefore, AOM/DSS murine models are important for the investigation of inflammation-induced colon carcinogenesis and chemoprevention strategy screening.

## 3. Molecular Mechanisms Associated with Chronic Inflammation and Colorectal Cancer

### 3.1. Inflammation-Dependent Oxidative Stress

Previous studies identified that carcinogenesis in IBD was related to reactive nitrogen intermediates (RNI) and reactive oxygen species (ROS) [3,25,26]. RNI and ROS are produced by neutrophils and macrophages, and they may cause oxidative damage to DNA, proteins, and lipids in the surrounding mucosal cells [3,23,24]. RNI, an indicator of nitrosative stress, is associated with nitrotyrosine, which is formed when peroxynitrite interacts with protein tyrosine. Hence, nitrotyrosine can serve as an important biomarker. In murine treated with AOM/DSS, more numerous and concentrated nitrotyrosine positive cells were observed as well as the greater expression of iNOS [18]. ROS are small oxygen-derived molecules produced by various biochemical and physiological oxidative processes, including superoxide (O_2_^−^), hydroxyl (OH), peroxyl (RO_2_), and alkoxyl (RO), hypochlorous acid (HOCl), ozone (O_3_), singlet oxygen (O_2_), and hydrogen peroxide (H_2_O_2_) [21]. The imbalance in the generation and elimination of ROS and RNI was found to lead to oxidative stress. Hepatic malondialdehyde (MDA) levels, which are indicators of oxidative stress, were increased in murine treated with AOM/DSS [27]. Enzymatic and nonenzymatic antioxidants, such as superoxide dismutase, catalase, glutathione peroxidase and glutathione reductase, were decreased in an AOM/DSS mouse model [27]. Deficiencies in antioxidant genes, such as transcription factor NF-E2-related factor 2 (Nrf-2) and glutathione peroxidase 3 (Gpx3), were associated with increased numbers of aberrant crypt foci and tumors with higher dysplasia [28,29].

In patients with IBD-associated CRC, increased RNI and ROS concentrations were related to oxidative damage and active DNA damage response at cancer sites [30,31]. In AOM/DSS mouse models, knocking down genes that code for DNA damage repair-related enzymes, e.g., Alkyladenine DNA glycosylase (Aag), mutY DNA glycosylase (Mutyh), 8-oxoguanine DNA glycosylase 1 (Ogg1), may increase CRC incidence [9,30,32]. Overall, there is sufficient evidence from animal studies to support the carcinogenic role of inflammation-dependent oxidative stress in CRC developed from IBD.

### 3.2. Genomic Instability

Genomic instability includes base pair mutation, microsatellite instability, chromosome abnormality, gene fusion, gene copy number change, and other small or large structural variations. Inflammation-dependent oxidative stress is also related to high genomic instability in IBD. Genomic instability profiles were found significantly different between IBD-related CRC and sporadic CRC [33]. For example, mutations in IDH1 R132, BRAF V600E; the amplification of FGFR1, FGFR2, ERBB2; and the fusion protein EML4-ALK are more commonly found in IBD related CRC compared to sporadic CRC. Microsatellite instability formed by the malfunction of genes coding for DNA repair mismatch enzymes was found to be a clinical feature of Lynch syndrome and sporadic CRC, but not IBD-related CRC [34]. Little overlap between microsatellite instability in IBD-related CRC and sporadic CRC has been found [35,36]. Aneuploidy is higher in IBD-related CRC than in sporadic CRC, and it could function as an independent factor for the prediction of dysplasia and CRC developed in IBD patients [25,37]. Due to the higher inflammation-dependent oxidative stress and genomic instability in IBD, the critical molecular events in CRC development in sporadic CRC differ from those in IBD-related CRC. For example, the loss of heterozygosity in chromosome 17p, the locus of TP53 (a well-known tumor suppressor) and mutations of TP53 occurred earlier in IBD-related CRC than in sporadic CRC [3,38]. Other study found that the frequency of TP53 mutation in inflamed, non-dysplastic colonic epithelia was correlated with increased oxidative stress [39]. TP53 mutation may be caused by oxidative stress and inflammation in IBD, which promote carcinogenesis of IBD-related CRC. Inflammation-dependent oxidative stress and genomic instability play critical roles in IBD-related carcinogenesis.

### 3.3. Cytokines

A pro-inflammatory microenvironment also contributes to carcinogenesis in IBD. Studies on IBD-associated CRC patients and AOM/DSS mouse models showed that activated neutrophils, fibroblasts, dendritic cells, macrophages, and T cells were present in IBD related CRC tissue [40,41,42,43]. The cytokines released by these effectors in a pro-inflammatory microenvironment also showed distinct roles in carcinogenesis in IBD. For instance, the tumor necrosis factor (TNF), which is released by monocyte/macrophage lineages and plays an important role in cell proliferation, differentiation and death, was shown to be increased in an AOM/DSS mouse model [44]. Hence, TNF-α antagonists could significantly reduce the number and size of tumors in this model [18,45]. Interleukin 1 (IL-1)/interleukin 6 (IL-6) axis activation was found to be involved in IBD related CRC carcinogenesis. In IBD, IL-6 is mainly released by monocytes, macrophages, and by T and B lymphocytes. IL-1 produced by neutrophils has also been shown to promote the development of CRC in IBD by inducing IL-6 production in intestine-resident mononuclear phagocytes [43]. High expressions of IL-1β and IL-6 were found in an AOM/DSS mouse model [18]. Knocking down IL-6 in AOM/DSS mouse models reduced the number of tumors developed [46,47]. The IL-23/IL-17 axis, including IL-17A, IL-17F, IL-22, and IL-23, are also important cytokines in IBD. The elevated IL-17A and IL-17F in IBD patients are released by Th17 cells. The successful development of Th17 cells to induce chronic inflammation was found to be dependent on the high levels of IL-21, IL-23, IL-6, and TGF [48,49]. IL-23 was found to be essential for the expression of TNFα, IL-6, and TNFγ in the pro-inflammatory microenvironment. These cytokines have the potential to bind to receptors or transcriptional factors in order to activate related signaling pathways to promote carcinogenesis.

### 3.4. NFκB

A key molecular component in the pro-inflammation molecular network is nuclear factor kappa B (NFκB). Under normal circumstances, NFκB dimers remain in the cytoplasm by binding to the specific inhibitors of NFκB (IκBs). Cell stimulation activates IκB kinase (IKK) subunits (α, β and γ) to induce the degradation of IκB proteins. The unbound NFκB then translocates to the nucleus and initiates transcription of several hundred target genes to promote the carcinogenesis of CRC. Lipopolysaccharide (LPS), and pro-inflammatory cytokines TNFα, IL-1, and CD40 ligand can activate the NFκB pathway. In inflammatory cells, NFκB is generally activated by reacting to bacteria, virus, and necrotic cell production as well as some inflammatory cytokines. The activation of NFκB could induce its downstream pathways including growth and survival signals as well as angiogenic factors. In malignant cells, NFκB reacts to inflammatory cytokine, growth, and survival signals and angiogenic factors released from inflammatory cells and regulate cell cycle related genes, thus, induce apoptosis inhibition effects and promote invasion and metastasis. Specifically, in AOM/DSS mouse models, studies found that in myeloid cells, IKK-β-driven NFκB promoted the production of cytokines that acted as growth factors in pre-malignant enterocytes. In enterocytes, IKK-β-driven NFκB was found to activate anti-apoptotic genes, thereby suppressing the apoptotic elimination of pre-neoplastic cells.

### 3.5. Microbiome

The alteration in composition and function of bacterial microbiota and fungal microbiota are considered as significant factors for IBD development [50,51]. The gut microbiome is linked to chronic inflammation in IBD [52]. The cytokines and increased ROS production induced by inflammation favors the outgrowth of some bacteria or kill others, and shape the gut microbiome. The gut microbiome plays an important role in the host immune system. Gut microbiome regulates the function of T help cell profile, CD4+ Treg cell, the production of several interleukins, such as (IL)-17A, IL-17F, IL-21, and IL-22. Compared to healthy people, IBD patients have more bacteria with inflammatory function and fewer bacteria with anti-inflammatory function [50,51,53,54]. Bacteria with anti-inflammatory effects such as *Faecalibacterium prausnitzii, Blautia faecis, Roseburia inulinivorans, Ruminococcus torques, and Clostridium lavalense* decreased and adhesion-invasive *Escherichia coli* with pro-inflammatory effects increased in IBD patients.

## 4. Chemoprevention of Colorectal Cancer in Inflammatory Bowel Disease with Anti-Inflammation Pharmaceuticals

One of the first therapies used to treat IBD is 5-ASA, which inhibits the production of cyclo-oxygenase and prostaglandin, thromboxane synthetase, platelet activating factor synthetase, and IL-1 to reduce the acute inflammatory response [5,52]. However, a systematic review and meta-analysis conducted by Velayos and colleagues that assessed 5-ASA for CRC chemoprevention showed only a slight preventative role of 5-ASA in CRC development [52]. The chemopreventive effects of corticosteroid are not clear, as toxicities from long-term use have limited its implementation as a chemopreventive agent [5]. A major trial that was conducted to assess the roles of immunomodulators, such as azathioprine, 6-mercaptopurine, and methotrexate, showed no significant protective effects on CRC [5]. Both azathioprine and 6-mercaptopurine can inhibit purine nucleotide synthesis, metabolism and the DNA/RNA production in immune effectors, and methotrexate can inhibit DNA/RNA production by competitively inhibiting dihydrofolate reductase during tetrahydrofolate synthesis. Thus, these drugs may inhibit DNA/RNA productions in other somatic cells and increase the risk of gene structure change and genomic instability. The side effects of immunomodulators have been associated with increased risks of lymphoma and non-melanomatous skin cancers, thus, limit the use of these medications in IBD patients in the clinical setting. NSAIDs, which inhibit the conversion of arachidonic acid to prostaglandins in the cyclo-oxygenase-2 pathway, have some chemoprevention effects on CRC. The U.S. Preventive Services Task Force (USPSTF) recommends aspirin for adults aged 50–59 to prevent CRC. However, most of the IBD patients are diagnosed in their 20 s and 30 s [55], and USPSTF didn’t recommend aspirin for adults younger than 50 to take aspirin for colon cancer prevention. The role of aspirin in chemoprevention of IBD related CRC is limited. Moreover, several pieces of evidence showed that NSAIDs promote IBD development [2]. Overall, agents that target inflammation have shown modest results in preventing CRC development in IBD patients.

The complexity of the inflammatory pathways and multiple processes involved in CRC carcinogenesis indicates that a multitude of targets exist; hence, new chemoprevention agents are needed to address this issue. The additive and synergistic activities of multiple bioactive phytochemicals in natural food products may have greater overall efficacy and safety in cancer prevention. Hence, in the following section, we discuss the chemoprevention effects of black raspberry and strawberry on CRC.

## 5. Chemoprevention of Colorectal Cancer in Inflammatory Bowel Disease with Berries

### 5.1. Efficiency of Strawberry and Black Raspberry in Cell Lines and Animal Models

The major investigational models and findings on the chemoprevention effects associated with strawberry and black raspberry are summarized in Table 1. The black raspberry extract showed anti-cancer activity in HT-29 colon cancer cells by regulating the cell cycle and apoptosis signaling pathways [56,57]. In the DSS-treated ulcerative colitis mouse model, the short-term black raspberry intervention reduced the degree of mucosal ulceration, suppressed the levels of pro-inflammation cytokines TNF-α and IL-1β, and inhibited the COX-2 and NFκB signaling pathways [58]. When the berry intervention was extended for a longer period of time, the number of macrophages and neutrophils infiltrating the colon tissue decreased and the hypermethylation of tumor suppressor genes increased [59]. In another model of ulcerative colitis using IL-10 knockout mice, a diet of 5% black raspberry was shown to significantly reduce ulceration in colonic mucosa and submucosa, which are associated with aberrant epigenetic dysregulation in the Wingless/Integrated (Wnt) signaling pathway [60].

In mouse epidermal JB6Cl41 cells, carcinogen BaP diol-epoxide (BPDE) treatment activated the activator protein 1 (AP-1), NFκB, and COX-2, which were then inhibited by the methanol extract of black raspberry [61,62]. In an AOM-induced aberrant crypt foci rat model, black raspberry diets significantly reduced the aberrant crypt foci multiplicity, total tumor multiplicity, and urinary 8-hydroxy-2′-deoxyguanosine (8-OHdG) levels [63]. A black raspberry diet was also significantly associated with reduced expression of COX-2 and pro-inflammatory cytokines TNF-α, IL-1, IL-6, and IL-10. Interestingly, when it was compared to the mechanism associated with tumor development in Apc1638+/− mice, the inhibition of β-catenin was found only in Apc1638+/− mice [64].

Strawberry extracts were shown to inhibit HT-29 proliferation by stimulating cell apoptosis and p21WAF1 [57,67]. Strawberries also showed significant anti-tumor effects on CRC cells CaCo-2 and HCT-116 [68,69]. In a gum acacia-induced IBD rat model, ethanolic extract of *Fragaria vesca*, a wild strawberry, decreased the disease activity index and lesion scores and increased antioxidant enzymes including myeloperoxidase, tissue catalase and superoxide dismutase [70]. In the AOM/DSS mouse model, 2.5%, 5%, or 10% (wt%) lyophilized strawberry significantly decreased tumor incidence, suppressed nitrosative stress, decreased the inflammation mediators TNF-α, IL-1β, IL-6, COX-2 and iNOS, and inhibited the phosphorylation of phosphatidylinositol 3-kinase (PI3K), Akt (Protein Kinase B), extracellular signal-regulated kinase (ERK), and NFκB [18].

In comparison to other berries, black raspberry and strawberry showed the potential for superior effects in CRC cell lines. In a study that compared the pro-apoptosis effects of six popularly consumed berries (blackberry, black raspberry, blueberry, cranberry, red raspberry, and strawberry), black raspberry and strawberry showed the most significant pro-apoptotic effects in CRC cell lines [57]. In another study that compared different berry extracts, strawberries showed the best anti-proliferation effects with an EC50 value of 20 ug/mL [68].

### 5.2. Efficiency of Black Raspberry in Clinical Studies

It is reported that a phase I study with black raspberry has been conducted in patients with CRC. The overall objective of this study is to identify measurable genetic and epigenetic biomarkers modulated by black raspberry in patients with CRC. This study demonstrates that 60 g of black raspberry powder per day for nine weeks significantly modulate the Wnt pathway through demethylating tumor suppressor genes SFRP2 and WIF1, and decrease the expression of β-catenin and E-cadherin. Black raspberry also decreases the expression of DNMT1, GM-CSF and IL-8 as well as the proliferation of biomarker Ki-67 and apoptosis [65,66]. The reports on the effects of strawberry on CRC or IBD are not available.

### 5.3. Major Bioactive Components in Berries 

**Figure 2:**
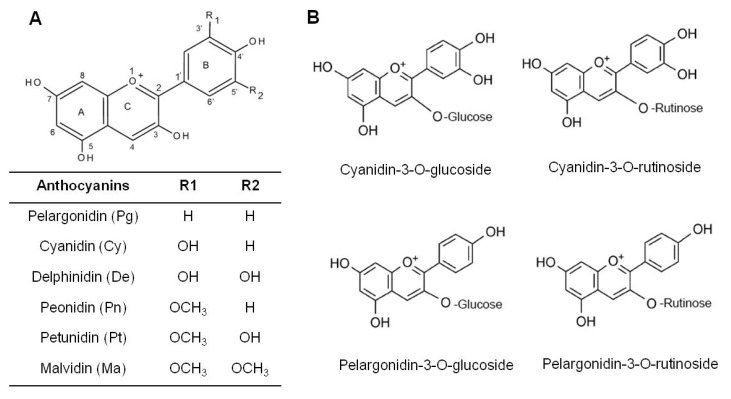
Structure of anthocyanins. (**A**) The structural classification of the six most common anthocyanins; (**B**) The structural classification of the four most common anthocyanins identified in strawberry and black raspberry.

Anthocyanins are flavonoid compounds that are responsible for the colors of most fruits and vegetables, and they contribute to the protective effects of fruits and vegetables against chronic diseases and cancer (Figure 2). According to our previous studies, anthocyanins are the main components in strawberry and black raspberry (Table 2) [18,22]. Among the anthocyanins, cyanidin rutinoside is the most abundant in black raspberry, whereas pelargonidin glucoside is the most abundant in strawberry. Pelargonidin rutinoside is the only anthocyanin that has been identified in both black raspberry and strawberry. Following anthocyanins, ellagic acid and derivatives are the second most abundant components of strawberry although they are rare in black raspberry. Among ellagic acid and its derivatives, agrimoniin is the main component of strawberry. Additionally, ellagitannins account for about 10% of the phytochemical components in black raspberry and strawberry. Flavonols have also been identified in both black raspberry and strawberry, but in much lower concentrations than anthocyanins.

Berry extracts and anthocyanin compounds have been studied both in vivo and in vitro. Table 3 provides a summary of the role of anthocyanins in CRC cell lines or animal models. Anthocyanin-enriched fractions from black raspberry inhibited cell growth in HT-29 and HCT-119 cells [57,67,69,71,72,73,74,75,76,77,78,79,80,81,82,83,84,85,86,87,88,89,90,91,92,93]. In CRC, an anthocyanin-enriched extract of black raspberry showed effects similar to black raspberry powder on CRC cell lines in suppressing cell proliferation, inducing apoptosis, decreasing the activity of DNMT1 and DNMT3B and of demethylate CDKN2A, SFRP2, SFRP5 and WIF1 in the Wnt pathway [60,71]. Cyanidin-3-glycoside, which is the anthocyanin found in black raspberry, decreased DNA strand breakage in human colon epithelial cells (HCEC), and it reduced cytotoxicity induced by peroxyl radicals by suppressing apoptosis and decreasing the sub-G1 phase of the cell population in Caco-2 CRC cells [72,73]. Cyanidin-3-*O*-beta glucopyranoside and its aglycon form, cyanidin chloride, was shown to have the potential to function in inhibiting cell growth and proliferation and in decreasing the ROS level in Caco-2 cells [74]. Strawberry extracts also showed cell growth and proliferation inhibition effects, antioxidative effects, and p21WAF1 suppression effects in HT-29, HCT-116 cells [57,66,70]. The anthocyanins identified in other fruit and vegetables that showed anti-cancer effects in CRC cells lines included the anthocyanins found in black raspberry and strawberry.

The distribution of anthocyanins has been found in almost all tissues. The black raspberry phytochemicals, cyanidin-3-rutinoside and cyanidin-3-xylosylrutinoside, have been detected in oral cancer tissues in patients after the administration of oral troches containing freeze-dried black raspberry powder as well as in prostate tissue in mice models after a diet of black raspberry powder [94,95]. Importantly, bioavailability and pharmacokinetic studies of strawberry anthocyanins showed that only modest amounts of ingested anthocyanins were absorbed from the upper small intestine. Most phytochemicals enter the colon where the substantial microbial metabolism and interaction with the colonic epithelium take place [18]. Protocatechuic acid (PCA) is one of the main metabolites of anthocyanins that can be absorbed by animals and humans. PCA significantly inhibited cell proliferation and colony formation in colon cancer SW 480 cells [75]. PCA also prevented diarrhea and bleeding in DSS-treated rats, and it decreased pro-inflammatory cytokines, nitric oxide concentration, and oxidative damage as well as the expression of COX-2 and iNOS [90]. Additionally, PCA diets may decrease the number of aberrant crypt foci, ornithine decarboxylase activity and the expression of AgNOR in AOM-induced CRC rat models [88,89]. In strawberries, 4-hydroxybenzoic acid (4HBA) was considered a metabolite of pelargonidin-3-glucoside, but it did not show anti-cancer activity in colon cancer in a limited number of studies [96,97].

The growth and spread of cancer depended not only on the biological characteristics of the tumor cells but also on the host immunology response. Black raspberry extracts and the single anthocyanins component cyanidin-3-rutinoside and quercitin-3-rutinoside were shown to inhibit T cell proliferation, limit myeloid-derived suppressor cells (MDSC) expansion, and suppress MDSC capacity [98]. Cyanidin-3-glucoside and cyanidin-3-rutinoside also reduced the inflammation cytokines TNF-α, IL-6, IL-1 β in lipopolysaccharide (LPS)-treated murine macrophage RAW264.7 cells [99].

In contrast to the consistent effects of anthocyanins in black raspberry and strawberry on chemoprevention, the results of studies that assessed the ellagitannin components of berries in chemoprevention have varied. Some studies found that ellagitannins were the most responsible for the anti-cancer activity of berry extracts [56,67]. However, the findings of other studies suggested that berry ellagitannins may not be sufficient for the prevention of many cancers, such as esophageal squamous cell carcinoma, because various concentrations of ellagitannins showed no differences in chemoprevention [100].

A recent study that used a high-resolution 1H NMR-based multivariate statistical model showed that anthocyanins, cyanidin 3-rutinoside, and cyanidin 3-xylosylrutinoside were the predominant contributors to the anti-cancer effects of black raspberry. However, in the same study, salicylic acid derivatives (salicylic acid glucosyl ester), quercetin 3-glucoside, quercetin 3-rutinoside, p-coumaric acid, epicatechin, methyl ellagic acid derivatives (methyl ellagic acetyl pentose), and citric acid derivatives were also shown to contribute significantly to anti-cancer effects [101].

Anthocyanins have brought the research community’s attention in the field of IBD [102,103]. In our opinion, anthocyanins are the major components responsible for the chemoprevention effects of black raspberry and strawberry. This opinion is based on five main lines of evidence: (1) anthoycanins are the most abundant components in black raspberry and strawberry; (2) the anti-cancer effects of anthocyanins and the role of anthocyanins in immune modification are well-documented; (3) PCA, the metabolites of anthocyanins in black raspberry, show chemoprevention effects similar to those of black raspberry; (4) anthocyanins are found in cancer tissues; (5) the colon is the major site of the metabolism of anthocyanins. However, because other components also significantly contribute to anti-cancer effects, it is clear that further studies are needed to assess the chemopreventive compounds in black raspberry and strawberry.

### 5.4. Mechanisms Associated with Preventative Effects of Berries on Colon Cancer

The benefits of berries in reducing antioxidative stress are well known. In a study that compared the antioxidant role of 10 phenolic compounds in strawberry extracts, the most potent antioxidants identified by a trolox equivalent antioxidant capacity (TEAC) assay were cyanidin-3-glucoside, cyanidin-3-pelargonidin, and kaempferol [66]. Moreover, urinary 8-OHdG levels and tissue nitrosative stress are important biomarkers of oxidative stress, which were reduced by the intake of black raspberry and strawberry [18,63]. Overall, several studies using cell lines, animal models, and human clinical trials of strawberry and black raspberry reported significantly decreased oxidative and inflammatory signals, such as COX-2 and NFκB. However, not all related signals were shown to be regulated by either black raspberry or strawberry, including the following: DNA repair-related enzymes, such as Aag, Mutyh, Ogg1; enzymatic and nonenzymatic antioxidants, such as SOD; and the antioxidant genes Nrf-2, Gpx3. Therefore, these molecular processes should be examined in future studies.

One form of genomic instability is the loss of heterozygosity, which in previous studies was reduced by the topical application of the black raspberry gel on oral intraepithelial neoplasia lesions (17p13 location containing TP53 genes are included in the loss of heterozygosity). Hypermethylation, or the increased number of methyl groups added to the promoter region, were shown to function to inhibit the transcription of target genes. Similar to the loss of heterozygosity, hypermethylation was shown to be an important approach in deactivating tumor suppressor genes during carcinogenesis. As previously mentioned in this review, black raspberry powder and anthocyanin-enriched extract of black raspberry worked to demethylate CDKN2A, SFRP2, SFRP5, and WIF1 in the Wnt pathway through suppressing DNMT1 and DNMT3B [60,71]. In a phase Ib study on the effects of black raspberries on rectal polyps in patients with familial adenomatous polyposis, a greater number of the demethylated transcription start sites and the increased expression of DNMT1 was found in patients who received a black raspberry intervention compared to those that did not [104]. Combined with the demonstrated antioxidant effects of black raspberry and strawberry, the above evidence suggests the potential of black raspberry and strawberry to prevent inflammation-dependent oxidative stress and genomic instability in colon epithelial cells.

In a DSS-treated ulcerative colitis mouse model, an intervention employing a long-term black raspberry diet resulted in the decreased infiltration by macrophages and neutrophils in colon tissue [61]. Black raspberry and strawberry were both associated with the reduced expression of COX-2 and the pro-inflammatory mediators, COX-2, iNOS, TNF-α, IL-1, IL-6, and IL-10 in cell studies, animal models, and clinical trials [17,18,19,20,21,22]. Cytokine GM-CSF and IL-8 were also found to be decreased by black raspberry powder in untreated colon cancer patients [67]. Black raspberry also altered innate immune cell trafficking in NMBA-induced esophageal squamous cell carcinoma in a rat model [105]. Single anthocyanins cyanidin-3-rutinoside and quercitin-3-rutinoside inhibited MDSC expansion and modulated T lymphocyte proliferation [98]. Hence, another interesting area for further research is the regulation of immune effectors by black raspberry and strawberry.

**Figure 3:**
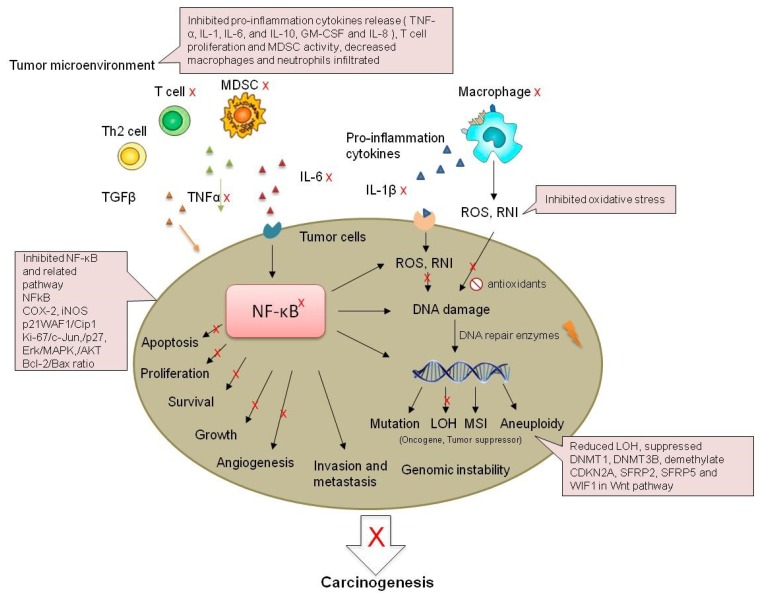
Possible mechanisms of the inhibition of IBD-related CRC by strawberry and black raspberry. MDSC: myeloid-derived suppressor cells; RNI: reactive nitrogen intermediates; LOH: Loss of heterozygosity; MSI: microsatellite instability.

NFκB is present in almost all cell types, and it is involved in inflammation, cell differentiation, and carcinogenesis. The cyclin-dependent kinase inhibitor, p21WAF1/Cip1, was induced by black raspberry in a Muc2−/− mice CRC model [64]. In addition, the cell proliferation markers Ki-67, c-Jun, p27, Erk1/2, MAPK, and AKT were regulated by treatment with black raspberry or strawberry [18,103,104,106]. Oxidative stress activates MAPK family members, including p38 MAPK and JNK, which subsequently activated genes involved in cellular proliferation. MAPK is activated by a range of stimuli, and it mediates several physiological processes, which are observed during carcinogenesis. AKT signaling plays an important role in multiple cellular processes, including cell proliferation, survival, motility, and angiogenesis. The induction of cell survival by AKT is mediated through NFκB signaling. The activation of the AKT and ERK pathways acts synergistically to promote the mechanistic target of rapamycin signaling, which controls NFκB activity [18,20]. NFκB was also found to regulate cell apoptosis during carcinogenesis. Black raspberry and strawberry increased apoptosis markers, TUNNEL staining, and the Bcl-2/Bax ratio in both animal models and human patients [16,105,106,107,108]. NFκB is required for the stabilization of snail, which is the transcription factor involved in regulating the expression of E-cadherin to induce cell migration and invasion induced by inflammatory cytokines. The inhibition of NFκB was found to potentially induce the degradation of Snail, which increased the expression of E-cadherin and hence inhibited cell migration and invasion. NFκB signaling was an important molecular event associated with the chemoprevention effects of black raspberry and strawberry on IBD-induced CRC. The mechanisms associated with the chemoprevention effects of black raspberry and strawberry are summarized in Figure 3.

## 6. Conclusions

Strawberry and black raspberry have been shown to have potential cancer prevention effects with low toxicity profiles in IBD-related CRC. Inflammation-induced carcinogenesis has been associated with oxidative stress, genomic instability, immune effectors, cytokine dysregulation, and the NFκB signaling pathway. In contrast to anti-inflammation pharmaceuticals, strawberry and black raspberry interventions have shown to have a synergistic role in multiple molecular events, including suppressing cytokines release, decreasing oxidative stress, reducing genomic instability, and inhibiting NFκB and related signaling pathways. The chemopreventive activity of strawberry and black raspberry is likely due to multiple nutrients and bioactives, especially anthocyanins. The clinical translational application of berries in IBD patients for CRC prevention is limited, which may suggest that this “low-hanging fruit” should be assessed in future clinical trials on colon cancer prevention in IBD. The evidence to date is a step toward the development of specific phytochemicals and metabolites as chemopreventive agents based on the principles of pharmacognosy.

## Figures and Tables

**Table 1:** Preventive effects of black raspberry and strawberry on Inflammatory Bowel Disease (IBD) and CRC.

Systems	Major Findings	Ref.
**Black Raspberry**		
**1. CRC Cell Lines**		
HT-29 /HT-116 cell lines	Regulating cell cycle and apoptosis	[56,57]
**2. Animal Models of IBD**		
DSS treated mouse	Colonic epithelium acute injury↓, ulceration↓, TNF-α and IL-1β↓, COX2 and NFκB↓	[58]
DSS treated mouse	Ulceration↓, macrophages and neutrophils infiltrated the colon tissue↓, NFκB↓, Dkk3↑, β-Catenin nuclear localization↓,c-Myc, DNMT3B, HDAC1, HDAC2, MBD2↓	[59]
IL-10 knockout mice	Ulceration↓, Wnt pathway↓, wif1, sox17, and qki↑,dnmt3b, hdac1, hdac2, and mbd2↓	[60]
**3. Animal Models of IBD-Related CRC**		
Mouse epidermal JB6Cl41 cells	AP-1, NFκB, and COX-2↓	[61,62]
AOM induced rat model	ACF multiplicity↓, total tumor multiplicity↓, urinary 8-OHdG↓	[63]
Muc2−/− mice	COX-2, TNF-α, IL-1, IL-6, and IL-10 ↓	[64]
**4.** **Clinical Studies of CRC**		
CRC patients	GM-CSF and IL-8↓, Ki-67↓, apoptosis↓	[65]
CRC patients	Wnt pathway↓ (SFRP2, WIF1, β-catenin, E-cadherin), DNMT1↓	[66]
**Strawberry**		
**1. CRC Cell Lines**		
HT-29 cell lines	Proliferation↓, cell apoptosis and p21WAF1↑	[57,67]
CaCo-2	Proliferation↓	[68]
HCT-116	Proliferation↓	[69]
**2. Animal Models of IBD**		
Gum acacia induced IBD rats	Disease activity index↓, lesion scores↓, antioxidant enzymes myeloperoxidase↑, tissue catalase↑, superoxide dismutase↑	[70]
**3. Animal Models of IBD-Related CRC**		
AOM/DSS mouse	Tumor incidence↓, nitrosative stress↓, TNF-α, IL-1β, IL-6, COX-2 and iNOS ↓, PI3K, Akt, ERK and NFκB↓	[18]

Note: ↓, decrease; ↑, increase.

**Table 2:** Major components in strawberry and black raspberry.

Strawberry			Black Raspberry		
	**mg/** **100 mg**	**% by** **mg**		**mg/** **100 mg**	**% by** **mg**
**Anthocyanins**			**Anthocyanins**		
pelargonidin glucoside	367.7	41.1	cyanidin rutinoside	2924.7	58.2
pelargonidin malonyl glucoside	83.9	9.4	cyanidin xylorutinoside	916.3	18.2
pelargonidin rutinoside	55.3	6.2	cyanidin glucoside	245.1	4.9
cyanidin glucoside	14.9	1.7	cyanidin sambubioside	103.5	2.1
			pelargonidin rutinoside	38.3	0.8
	**130.4**	**58.4%**		**4227.9**	**84.2%**
**Ellagitannins**			**Ellagitannins**		
ellagitannin	64.1	7.2	sanguiin H6	173.2	3.4
ellagitannin	11.4	1.3	ellagitannin 783-1	101.1	2.0
ellagitannin	23.1	2.6	ellagitannin 933-2	75.8	1.5
Lambertianin	20.3	2.3	elagitannin 783-2	75.8	1.5
			ellagitannin 935-1	62.8	1.3
			ellagitannin 933-1	50.5	1.0
			Lambertiannin	31.3	0.6
			ellagitannin 935-2	7.9	0.2
	**118.9**	**13.3%**		**578.5**	**11.5%**
**Ellagic acid and derivatives**			**Ellagic acid and derivatives**		
Agrimoniin	144.5	16.2	methyl ellagic acid pentoside	16.0	0.3
ellagic acid rhamnoside	23.1	2.6	ellagic acid	9.3	0.2
ellagic acid	7.3	0.8	ellagic acid rhamnoside	5.8	0.1
			myricetin hexoside, EA derivative (coelution)	3.8	0.1
	**174.9**	**19.5%**		**34.8**	**0.7%**
**Flavonols**			**Flavonols**		
quercetin hexuronide	58.8	6.6	quercetin hexuronide	82.2	1.6
kaempferol glucoside/hexuronide	14.5	1.6	rutin (quercetin rutinoside)	75.4	1.5
kaempferol malonyl hexoside	5.1	0.6	quercetin xylorutinoside	24.2	0.5
	**78.4**	**8.8%**		**181.8**	**3.6%**

**Table 3:** Effects of anthocyanins in IBD and IBD-related CRC.

Original Sources	Models	Major Findings	Anthocyanin Profiles	Ref.
**Cell Lines**				
**1. Black Raspberry**				
Black raspberry extract	Cell line HT-29 HCT-116	Inhibited cell growth HT-29 IC50 = 89.11, HCT-116, IC50 = 89.00	cyanidin-3-sophoroside rhamnoside, cyanidin-3-sambubioside rhamnoside, cyanidin-3-rutinoside	[57]
Black raspberry anthocyanin-enriched extract	Cell line HCT116	Decreased DNMT activity; decreased methylation of CDKN2A, SFRP5, SFRP2 and WIF1; suppressed cell proliferation; induced apoptosis	cyanidin-3-*O*-glucoside, cyanidin- 3-*O*-rutinoside, cyanidin-3-*O*-xylosylrutinoside, cyanidin-3-*O*-sambubioside	[58]
**2. Strawberry**				
Strawberry extract	Cell line HT-29 HCT-116	Inhibited cell growth HT-29 IC_50_ = 114.30, HCT-116, IC_50_ = 62.00	cyanidin-3-glucoside, pelargonidin-3-glucoside, pelargonidin-3-rutinoside	[57]
Strawberry extract	Cell line HT29 HCT-116	Antioxidative effects.	cyanidin-3-glucoside, pelargonidin, cyanidin-3-glucoside, pelargonidin, pelargonidin-3-rutinoside	[66]
Strawberry extract	Cell line HT29	Inhibited proliferation; reduced expression of p21WAF1	cyanidin derivative; pelargonidin derivative	[67]
**3. Anthocyanins**				
cyanidin-3-glycoside	Cell line HCEC	Decreased DNA strand breakage	cyanidin-3-glycoside	[72]
Cyanidin-3-glycoside	Cell line Caoco-2	Reduced cytotoxicity induced by AAPH; suppressed apoptosis; decreased sub-G1 phase cell population	cyanidin-3-glycoside	[73]
Cyanidin-3-O-beta glucopyranoside, cyanidin chloride	Cell line Caoco-2	Inhibited cell growth and proliferation; decreased reactive oxygen species (ROS) level	cyanidin-3-*O*-beta glucopyranoside, cyanidin chloride	[74]
**4. Protocatechuic Acid (PCA)**				
Brown rice extracted PCA	Cell line SW480	Inhibited cell growth and colony formation	PCA	[75]
**5. Other Fruit and Vegetables**				
Blueberry	Cell line Caoco-2	IC_50_ 0.53 ± 0.04	delphinidin 3-galactoside, delphinidin 3-glucoside, cyanidin 3-galactoside, delphinidin 3-arabinoside, cyanidin 3-glucoside, petunidin 3-galactoside, cyanidin 3-arabinoside, petunidin 3-glucoside, peonidin 3-galactoside, petunidin 3-arabinoside, peonidin 3-glucoside, malvidin 3-galactoside, peonidin 3-arabinoside, malvidin 3-glucoside, malvidin 3-arabinoside	[76]
Blueberry extract	Cell lines HT-29	Inhibited cell growth; induced apoptosis	delphinidin 3-*O*-β-glucopyranoside; cyanidin 3-*O*-β-galactopyranoside; cyanidin 3-*O*-β-glucopyranoside; petunidin 3-*O*-β-glucopyranoside; peonidin 3-*O*-β-galactopyranoside; peonidin 3-*O*-β-glucopyranoside; malvidin 3-*O*-β-glucopyranoside.	[77]
Bilberry purified anthocyanins	Cell line HCT-116	Decreased cell viability	pelargonidin, cyanidin, peonidin, delphinidin, and malvidin	[78]
Cocoplum anthocyanins exert	Cell line HT-29	Cell proliferation was suppressed; increased intracellular ROS production; increased intracellular ROS production	delphinidin-3-glucoside, cyanidin 3-glucoside, petunidin 3-glucoside, delphinidin 3-(6-acetoyl) galactoside, delphinidin 3-(6-oxaloyl) arabinoside, peonidin 3-glucoside, petunidin 3-(6-acetoyl) galactoside, petunidin 3-(6-oxaloyl) arabinoside, peonidin 3-(6-acetoyl) glucoside, peonidin 3-(6-oxaloyl) arabinoside	[79]
Eugenia jambolana (Java Plum) fruit extract	Cell lines HCT-116 colon cancer stem cells	Inhibited proliferation; induced apoptosis	delphinidin-3,5-diglucoside, cyanidin-3,5-diglucoside, ptunidin-3,5-diglucosid, dtunidin-3,5-diglucosid, peonidin-3,5-diglucoside, monidin-3,5-diglucosid, cyanidin-3-glucoside, petunidin-3-glucoside, etunidin-3-glucosi	[80]
Anthocyanin-containing baked purple-fleshed potato extract	Colon cancer stem cells	Suppressed proliferation; elevated apoptosis; decreased β-catenin, c-Myc and Cyclin D1	pet-3-rut-5-glc, mal-3-rut-5-glc, cya-3-*O*(6-*O*-malonyl- β-d-glc), peo-3-(p-coum)-isophoro-5-glc, peo-3-rut-5-glc, pet-3-(p-coum)-rut-5-glc, peo-3-caffeyl-rut-5-glc, pel-3-(p-coum)-rut-5-glc, pel-3-(4-ferul-rut)-5-glc, peo-3-(p-coum)-rut-5-glc, mal-3-(p-coum)-rut-5-glc	[81]
Blue maize extract	Cell line Caco2 and HT29	Suppressed proliferation	cyanidin 3-glucoside, cyanidin 3-glucoside, cyanidin malonyl-glucoside, cyanidin succinyl-glucoside, pelargonidin 3-glucoside, pelargonidin malonyl-glucoside	[82]
Eggplant extract	Cell lines HT-29	Decreased DNA damage	delphinidin-3-rhamnosyl-glucoside-5-glucoside, delphinidin-3-rutinoside-5-glucoside	[83]
anthocyanin-enriched purple-fleshed sweet potato	Cell line SW480	Decreased cell number, G1 phase arrest	peonidin-3-glucoside	[84]
Vitis coignetiae Pullia extract		Inhibited cell invasion; suppressed MMP-2, MMP-9, NFkB	delphinidin-3,5-diglucoside, cyanidin-3,5-diglucoside, petunidin-3,5-diglucoside, delphinidin-3-glucoside, malvdin-3,5-diglucoside, peonidin-3,5-diglucoside, cyanidin-3-glucoside, petunidin-3-glucoside, peonidin-3-glucoside, malvidin-3-glucoside	[85]
Blackberry extract	Cell lines HT-29	Inhibited cell growth; inhibited IL-12 release	cyanidin-3-glucoside, cyanidin-3-arabinoside, delphinidin-3-xyloside, cyanidin-3-xyloside, cyanidin-3-malonylglucoside, cyanidin-3-dioxalylglucoside	[86]
Tart cherry anthocyanin	Cell line HT 29, HCT16	Inhibited cell growth	3-cyanidin 2″-*O*-β-d-glucopyranosyl-6″-*O*-α-l-rhamnopyransyl-β-d-glucopyranoside	[87]
**Animal Models**				
**1. PCA**				
PCA	AOM-treated rat	Decreased the number of aberrant crypt foci, ornithine decarboxylase activity and AgNOR	PCA	[88,89]
PCA	DSS-treated rat	Prevented diarrhea and bleeding; decreased pro-inflammatory cytokines; nitric oxide concentration, oxidative damage, and expression of COX-2 and iNOS	PCA	[90]
**2. Other fruit and vegetables**				
Anthocyanin-rich extracts from bilberry, chokeberry, and grape	AOM-treated rat	Reduced total ACF and the number of large ACF Suppressed cell proliferation reduces COX-2		[91]
Anthocyanin derived from purple sweet potato color in their basal diet	AOM/DSS rats	Decreased MDF, colon weight, low-grade dysplasia and total histopathology changes; decreased the expression of β-catenin, Ki67and Cyclin D1		[92]
anthocyanin-enriched purple-fleshed sweet potato	Animal AOM mice	Suppressed formation of aberrant crypt foci; decreased PCNA; increased caspase-3	peonidin-3-glucoside	[84]
Tart cherry anthocyanin	Apc^Min^ mice	Decreased the number and volume of adenomas	3-cyanidin 6″-*O*-α-l-rhamnopyranosyl-β-d-glucopyranoside	[87]
Tomato	DSS mice	Increased bacterial *Parabacteroides* and *Lactobacilli*		[93]
